# Structural Insights into the Interaction of Bisphenol F (BPF) and Bisphenol S (BPS) with Estrogen Receptors for Endocrine Safety Assessment

**DOI:** 10.3390/toxics14030262

**Published:** 2026-03-17

**Authors:** Ishfaq Ahmad Sheikh, Irshad Ul Haq Bhat, Torki A. Zughaibi, Mohamed A. Ghorab, Mohd Rehan, Majid Farhan Almutairi, Mohd Amin Beg, Zainab Tariq, Abdel Rezak M. Kadry

**Affiliations:** 1 Toxicology and Forensic Sciences Unit, King Fahd Medical Research Center, King Abdulaziz University, Jeddah 21589, Saudi Arabia; 2 Department of Medical Laboratory Sciences, Faculty of Applied Medical Sciences, King Abdulaziz University, Jeddah 21589, Saudi Arabia; 3 Department of Chemistry, College of Science, University of Bahrain, Zallaq 32038, Bahrain; 4 Wildlife Toxicology Lab, Department of Animal Science, Institute for Integrative Toxicology (IIT), Michigan State University, East Lansing, MI 48824, USA; 5 EcoHealth Unit, King Fahd Medical Research Center, King Abdulaziz University, Jeddah 21589, Saudi Arabia; 6 Diriyah Hospital, Ministry of Health, Riyadh 13717, Saudi Arabia; 7 Department of Environmental Sciences, Faculty of Engineering, Jamia Millia Islamia, New Delhi 110025, India; 8 Department of Global, Environmental, and Occupational Health (GEOH), University of Maryland School of Public Health, College Park, MD 20742, USA

**Keywords:** bisphenol F, bisphenol S, estrogen receptor, structural binding characterization, endocrine-disrupting chemicals, safety assessment

## Abstract

Endocrine-disrupting chemicals (EDCs) perturb hormonal homeostasis, dysregulating multiple biological pathways and subsequently resulting in adverse health outcomes, including impaired reproductive function. Bisphenols represent an important subclass of EDCs with widespread use in polycarbonate plastics, thermal paper formulations, epoxy resins, and various everyday consumer products. Bisphenol A (BPA) was the first bisphenol to be synthesized, with extensive industrial applications. However, the concerns over its potential health risks, most notably reproductive dysfunction, prompted the development and introduction of several structurally related BPA analogues. That said, studies on the potential hormonal effects of these BPA analogues remain limited. Therefore, strengthening the evidence base on their reproductive safety evaluation remains an essential priority for ensuring their safe application, and this study contributes to that broader objective. The study aimed to explore the potential endocrine-disrupting activity of two commonly used BPA analogues, bisphenol F (BPF) and bisphenol S (BPS), on reproductive hormone signalling, contributing to ongoing safety assessment efforts. The molecular interactions of these analogues with the estrogen receptor-α (ERα) and estrogen receptor-β (ERβ) were analyzed through structural binding characterization employing the induced fit docking (IFD) approach using the Schrödinger 2019 suite. The overall results revealed that the two indicated BPA analogues were placed successfully in the ligand-binding pockets of ERα and ERβ. Their binding pattern and molecular interactions showed certain similarities; however, they did not fully replicate the amino acid residue environment of the native ligands of ERα and ERβ, estradiol. Notably, the binding energy estimations revealed that BPF and BPS showed substantially lower values than those calculated for native ligands of ERα and ERβ. In summary, this study suggests that BPF and BPS exhibit lower predicted binding affinity toward ERα and ERβ under the applied molecular docking conditions. However, these computational findings do not establish receptor activation, endocrine potency, or safety outcomes, and the potential involvement of other reproductive signalling pathways warrants further investigation.

## 1. Introduction

Endocrine-disrupting chemicals (EDCs) perturb hormonal homeostasis through mechanisms that extend beyond single molecular targets, affecting endocrine regulation at multiple critical control points [1,2,3,4]. These compounds may function as hormone mimetics or antagonists at nuclear and membrane-associated receptors, modulate biosynthetic and metabolic pathways, interfere with carrier protein function, or dysregulate feedback mechanisms within major endocrine axes, including the hypothalamic–pituitary–thyroid and hypothalamic–pituitary–gonadal systems [5]. Critically, authoritative assessments have established that numerous EDC-mediated effects manifest at environmentally relevant exposure concentrations and frequently exhibit nonmonotonic dose–response relationships, challenging conventional toxicological paradigms [6,7]. Convergent evidence across cellular, tissue, and population-based investigations demonstrates that EDCs operate through interconnected mechanistic pathways encompassing receptor-mediated signal transduction, oxidative stress responses, and immune system modulation, with significant implications for disease susceptibility and progression [8,9,10]. Consistent with these mechanisms, extensive research has documented associations between EDC exposure and multiple physiological disorders [11,12,13,14,15]. Among these health endpoints, reproductive system disorders merit particular attention given their heightened sensitivity to endocrine disruption, underscoring the imperative for comprehensive toxicological evaluation [16,17,18].

Bisphenols represent a critical chemical class within the broader EDC landscape, with profound implications for human health that exemplify many cardinal concerns associated with endocrine disruption [19,20]. Bisphenol A (BPA), the most extensively characterized member of this class, possesses structural features conducive to hormone receptor interactions. Its widespread industrial applications in polycarbonate plastics, epoxy can linings, and thermal receipt paper result in ubiquitous human exposure, as evidenced by biomonitoring data demonstrating near-universal detection across human populations [21,22]. The presence of nonmonotonic dose–response profiles and heightened developmental sensitivity complicates conventional risk assessment approaches and raises concerns that even transient exposures during critical windows may produce persistent alterations in endocrine regulation [5,6]. BPA demonstrates binding affinity for both classical nuclear estrogen receptors and membrane-associated estrogen receptors, eliciting genomic and rapid non-genomic signalling responses at environmentally relevant concentrations [6,23]. However, BPA’s endocrine activity extends beyond estrogenic pathways to include androgen receptor antagonism and thyroid hormone transport disruption, contributing to diverse adverse health outcomes with particular relevance to reproductive dysfunction [5,22,24]. Given the fundamental role of estrogen receptor signalling in reproductive physiology, its perturbation by EDCs can precipitate serious reproductive health consequences, including infertility and hormonal dysregulation, emphasizing the critical importance of assessing potential long-term ramifications of such disruptions [25].

In response to escalating regulatory scrutiny of BPA driven by accumulating toxicological evidence, multiple structurally analogous substitutes marketed under “BPA-free” designations have been introduced into consumer products. Two prominently employed substitutes are bisphenol F (BPF) and bisphenol S (BPS) [2,26]. These BPA alternatives are extensively utilized in thermal papers, resin formulations, and food-contact materials, with biomonitoring data consistently confirming human exposure, particularly elevated BPS concentrations among populations with frequent thermal receipt handling [21,27,28,29,30,31,32]. While these analogues retain the fundamental bisphenol architecture, two aromatic rings connected through short alkyl chains or alternative chemical linkages [33,34], “BPA-free” labelling does not inherently ensure endocrine safety [5,31]. Furthermore, in contrast to BPA, these alternative bisphenols were not subjected to a comprehensive toxicological assessment prior to their commercial deployment [35]. Consequently, despite increasing concern, the evidence base characterizing BPS and BPF remains substantially less developed compared to BPA. Although available investigations have suggested potential BPF and BPS engagement with estrogen, androgen, and thyroid signalling pathways [31,36], the data remain limited, methodological approaches vary considerably, and longitudinal human studies are notably absent. This evidentiary gap carries significant implications because endocrine systems demonstrate responsiveness to low-level exposures through nonlinear mechanisms, indicating that restricted testing paradigms may fail to detect critical effects occurring exclusively within specific dose ranges or developmental windows [6]. Therefore, existing safety evaluation data regarding the endocrine-disrupting potential of BPF and BPS remain inconclusive, highlighting the urgent need for additional research to comprehensively assess their toxicological risks.

This scientific evidence underscores the necessity for comprehensive safety evaluations of BPA substitutes, particularly BPF and BPS, to elucidate their toxicological profiles and associated health risks. Strengthening the empirical foundation for reproductive safety assessment is essential to ensure appropriate use of these compounds, particularly given ongoing exposure concerns and mechanistic uncertainties. This investigation focuses on the potential endocrine-disrupting effects of two widely utilized BPA analogues, BPF and BPS, specifically examining estrogen receptor (ER)-mediated reproductive hormone signalling, a pivotal pathway in reproductive physiology. Disruption of ER signalling is well-established to precipitate significant reproductive dysfunction, underscoring its mechanistic importance. While potential involvement of additional reproductive signalling pathways cannot be entirely excluded, this study specifically addresses ER-mediated mechanisms, as a comprehensive evaluation of multiple reproductive targets within a single investigation is not feasible. Notably, existing studies examining the structural binding characteristics of BPF and BPS with estrogen receptors demonstrate inconsistency and yield inconclusive findings, highlighting a critical knowledge gap and the imperative for additional research. In light of conflicting reports regarding the effects of BPA substitutes on reproductive function, we have undertaken this computational investigation focusing on the estrogen receptor, a central mediator of reproductive endocrine signalling.

## 2. Materials and Methods

The structural binding characterization of the commonly used BPA analogues, BPF and BPS, within the ligand-binding pocket of ERα and ERβ was performed using the computational tools available in Schrödinger 2019, with Maestro 11.4 as the graphical user interface (Schrödinger, LLC, New York, NY, USA). This platform was utilized to analyze the binding modes of the ligands and to examine key molecular interactions within the receptor cavity, including hydrogen bonds, hydrophobic contacts, and other non-covalent interactions. In addition, binding energy estimations were conducted to evaluate the relative interaction strength of BPF and BPS with ERα and ERβ. The integrated computational workflow enabled consistent modelling, visualization, and comparative assessment of ligand–receptor interactions under standardized conditions. The ligand preparation protocol has been outlined in earlier studies [37]. The PubChem Compound ID (CID) of BPF and BPS are 12111 and 6626 respectively, and their Chemical Abstracts Service Registry Number (CASRN) are 620-92-8 and 80-09-1 respectively.

### 2.1. Protein Preparation

The protein data bank (PDB; http://www.rcsb.org/) was searched for three-dimensional crystal structural coordinates of human ERα and ERβ. The structure retrieved for human ERα was the crystal structure in complex with ligand estradiol with 2.40 Å resolution (PDB code: 1GWR). Similarly, the structure retrieved for human ERβ was the crystal structure in complex with ligand estradiol with 2.5 Å resolution (PDB code: 5TOA). Both structures were imported into the Glide docking module of Schrödinger 2019 (Schrödinger, LLC) and further processed using the Protein Preparation Wizard workflow, as previously reported [37]. The preparation protocol was applied to obtain chemically accurate and computationally reliable protein models prior to docking. During this procedure, crystallographic water molecules were removed, and missing hydrogen atoms were added to ensure correct protonation states and proper hydrogen-bonding capability. Appropriate atomic charges were assigned according to the selected force field parameters. Residues exhibiting multiple occupancies or missing atoms were carefully identified and corrected to maintain structural integrity. The hydrogen-bonding network was subsequently optimized by adjusting the orientations and protonation states of key polar residues, followed by restrained energy minimization to eliminate steric clashes while preserving the overall crystallographic conformation.

### 2.2. Ligand Preparation

**Figure 1:**
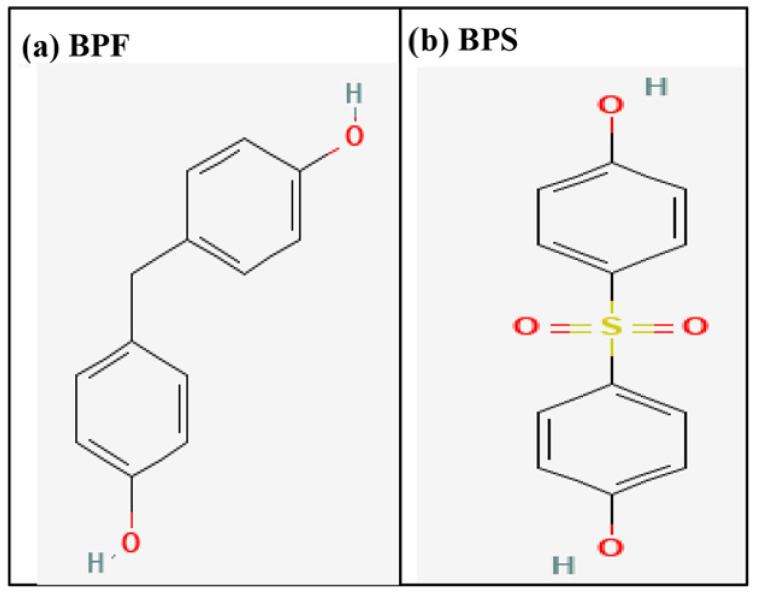
Two-dimensional structure of BPA analogues: BPF and BPS.

The three-dimensional structural coordinates of two commonly used bisphenols, BPF and BPS, were downloaded from the PubChem compound database (https://pubchem.ncbi.nlm.nih.gov/, accessed on 12 March 2026). These ligand structures were prepared for downstream experiments employing the LigPrep module (Schrödinger 2019–4: LigPrep, Schrödinger, LLC), as previously described [37]. This module operates with remarkable efficiency, processing nearly one ligand per second for downstream computational tasks. LigPrep refines the ligand by correcting structural inaccuracies and ensuring a proper Lewis structure, then generates a precise three-dimensional model through energy minimization. Ligands that fail to satisfy user-defined parameters are automatically excluded, while a diverse set of conformations, stereoisomers, tautomers, and ionization states are systematically produced from the input structure. The tautomers and protonation states are generated using Epik by predicting the most likely molecular forms at pH 7.0 ± 2, accounting for proton shifts, tautomeric equilibria, and microstate populations. Similarly, the estrogen receptor native ligand, estradiol, was also subjected to preparation for downstream computational tasks using LigPrep tool from Schrödinger Suite (2019 version). The 2D structures of the indicated BPA analogues, BPF and BPS, downloaded from the PubChem database, are presented in Figure 1.

### 2.3. Induced Fit Docking

The molecular interactions of BPF and BPS within the ligand-binding pocket of ERα and ERβ were performed through induced fit docking (IFD) using Schrödinger suite 2019-4 to account for both ligand flexibility and local protein side-chain rearrangements. In IFD, flexibility is induced both in the ligand and the binding pocket residues of the target protein rather than relying on a rigid docking approach, making the simulation more relevant to real-time conditions. The ligand protonation states and tautomer generated by LigPrep were subjected to geometry optimization using the OPLS3e force field. Grid was generated around the estradiol in the ligand-binding pocket, the native ligand of ERα and ERβ, using the default grid box settings (inner box ~10 × 10 × 10 Å^3^) and refinement parameters, applied consistently across all docking conditions.

Following initial docking, residues within ~5 Å of the ligand poses were sampled for conformational flexibility, allowing side-chain movements to relieve steric clashes and accommodate different ligand poses. Amino acid side-chain prediction and energy minimization were performed for both the ligand and receptor in each pose. The resulting protein–ligand complexes were then re-docked using Glide in extra precision (XP) mode with default settings, and the IFD score was estimated. Default IFD parameters, including standard van der Waals scaling for both ligand and receptor during the initial docking step, were applied consistently across all ligands, with no additional restraints on flexible residues unless specifically noted. The resulting complexes represent energetically favourable combinations of ligand conformations and side-chain adjustments, capturing both ligand flexibility and local protein adaptability while maintaining general applicability to all docking conditions. Finally, constrained minimization was applied to ERα and ERβ complexes to further refine the binding geometries. A thorough description of the IFD methodology can be found in our previous work [37]. Estradiol was also subjected to IFD in the ligand-binding pocket of ERα and ERβ.

### 2.4. Binding Affinity Calculations

The binding affinity estimation is an essential parameter in structural binding characterization, describing the strength of molecular interactions between a ligand and the target molecule. The binding affinity estimation of BPF and BPS for ERα and ERβ was estimated using the molecular mechanics generalized Born surface area (MM/GBSA) tool of Schrödinger 2019, as described previously [37]. MMGB-SA is a post-processing technique widely used to estimate the relative binding free energies of protein–ligand complexes. The method combines molecular mechanics (MM) energies with an implicit solvent model based on the Generalized Born (GB) approximation and a surface area (SA) term to account for nonpolar solvation effects. In general, the binding free energy (ΔG_bind) is calculated as the difference between the free energy of the protein–ligand complex and the sum of the free energies of the isolated protein and ligand. These free energies include contributions from bonded and non-bonded interactions (such as van der Waals and electrostatic terms), as well as solvation effects. Within the Schrödinger framework, the MM/GBSA calculation is typically performed after molecular docking or structural optimization. The complex is subjected to energy minimization to relieve steric clashes and optimize local geometry before energy evaluation. The resulting ΔG_bind values provide an estimate of the relative binding affinities of the ligands toward ERα and ERβ. Although MM/GBSA does not provide absolute experimental binding energies, it is commonly used for comparative ranking of ligands due to its balance between computational efficiency and reasonable accuracy.

## 3. Results

**Figure 2:**
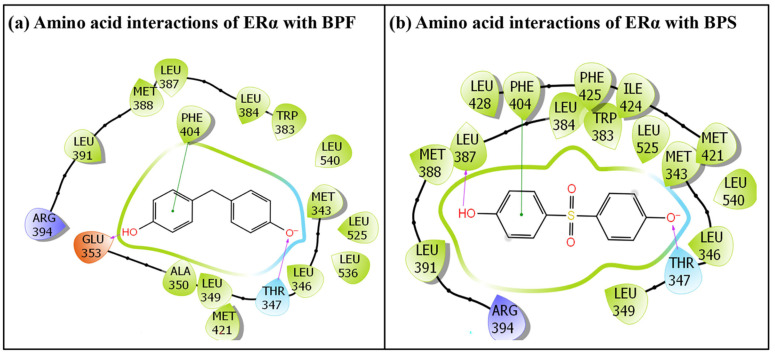
The molecular interactions of BPA analogues: (**a**) BPF, (**b**) BPS with amino acid residues lining the ERα ligand-binding pocket.

The commonly used BPA analogues, BPF and BPS, were placed into the ligand-binding pocket of ERα, suggesting successful docking of the given ligands. The IFD simulation experiment generated multiple poses for these docking complexes; however, only the best docking poses identified for both BPA analogue ligands, BPF and BPS, were selected for subsequent structural binding characterization analysis. Likewise, the ERα native ligand was successfully positioned into the ligand-binding pocket of ERα, suggesting successful docking of the ERα native ligand, estradiol. In this case as well, the best ranking pose was carried forward for further analysis of the native ligand. The chosen poses illustrating multiple amino acid residue interactions evaluated for both the BPA analogues, BPF and BPS, are shown in Figure 2. Both BPF and BPS interacted with 17 amino acid residues lining the ERα ligand-binding pocket; however, only 14 interacting amino acid residues were common between BPF and BPS (Figure 2). Binding poses were evaluated using the induced fit docking (IFD) workflow followed by MM-GBSA rescoring in Schrödinger 2019. In IFD, ligand poses are generated and refined through receptor side-chain and, when necessary, backbone adjustments to accommodate binding. Pose ranking is based on a composite score that considers both the docking score of the ligand within the refined receptor and the energetic contribution from receptor conformational changes. Selected poses were further assessed using MM-GBSA calculations, which estimate relative binding free energies by combining molecular mechanics energies with an implicit solvent model, accounting for bonded and non-bonded interactions as well as solvation effects. Pose selection criteria were based on favourable IFD scores, more negative MM-GBSA binding energies, chemically reasonable ligand conformations, and absence of steric clashes, ensuring physically and chemically plausible binding orientations.

**Figure 3:**
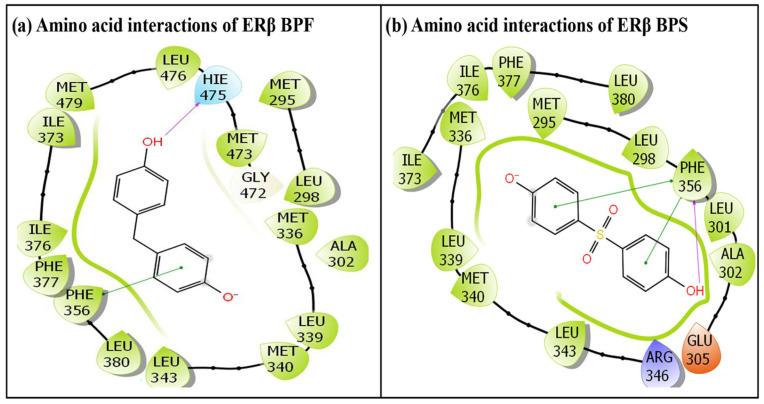
The molecular interactions of BPA analogues: (**a**) BPF, (**b**) BPS with amino acid residues lining the ERβ ligand-binding pocket.

**Figure 4:**
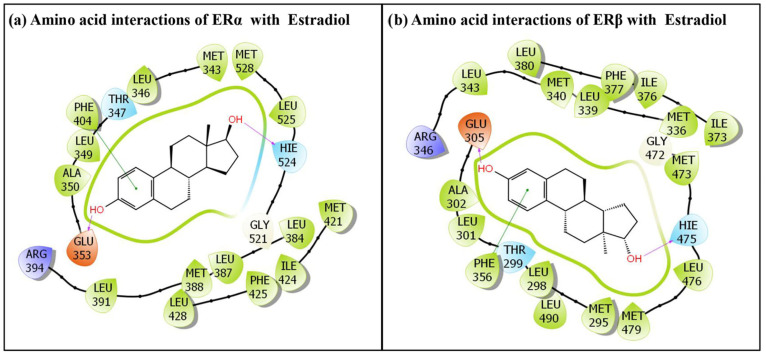
The molecular interactions of the estrogen native ligand, estradiol, with (**a**) amino acid residues lining the ERα ligand-binding pocket, and (**b**) with amino acid residues lining the ERβ ligand-binding pocket.

In the same way, the BPA analogues BPF and BPS were successfully accommodated within the ERβ ligand-binding pocket, indicating effective docking of these ligands. The IFD simulation generated multiple docking poses for each complex; however, only the top-ranked poses for BPF and BPS were selected for subsequent structural characterization. Figure 3 presents the selected poses, highlighting the key amino acid interactions evaluated for both the BPA analogues, BPF and BPS. Similarly, the docking pose of the ERα and ERβ native ligand, estradiol, is depicted in Figure 4. The native ligand, estradiol, was likewise positioned correctly within the binding pocket of ERα and ERβ, confirming successful docking, and its highest-scoring pose was retained for further analysis. The Figure 4a represents the native docking pose of estradiol in the ERα binding pocket, while Figure 4b represents its native docking pose in the ERβ binding pocket. In total, BPF interacted with 17 amino acid residues within the ERβ binding pocket (Figure 3a), whereas BPS engaged with 15 amino acid residues (Figure 3b), with 12 interacting amino acid residues common between BPF and BPS.

### 3.1. IFD of Bisphenols with ERα

#### 3.1.1. IFD of BPF with ERα

The BPF-ERα docking complex demonstrated extensive interactions with multiple ERα amino acid residues. In total, 17 residues participated in molecular interactions with BPF, including hydrogen bonds, van der Waals forces, and hydrophobic interactions. The interacting residues were Met-343, Leu-346, Thr-347, leu-349, Ala-350, Glu-353, Trp-383, Leu-384, Leu-387, Met-388, Leu-391, Arg-394, Phe-404, Met-421, Leu-525, Leu-536 and Leu-540. Two hydrogen-bonding interactions with Glu-353 and Thr-347 were observed. In addition, one pi–pi interaction with Phe-404 was also observed (Figure 2a).

Similarly, the molecular interactions between the native ligand estradiol and ERα are depicted in Figure 4a. A total of 20 ERα residues were involved in interactions with estradiol, including Met-343, Leu-346, Thr-347, leu-349, Ala-350, Glu-353, Leu-384, Leu-387, Met-388, Leu-391, Arg-394, Phe-404, Met-421, Ile-424, Phe-425, Leu-428, Gly-521, HIE-524, Leu-525 and Met-528. Two hydrogen-bonding interactions with Glu-353 and Hie-524 were observed. In addition, one pi–pi interaction with Phe-404 was also observed (Figure 4a).

Additional docking metrics, including the IFD score, Dock score, and Glide score, which are important for evaluating the structural binding behaviour of BPA analogue, BPF and the estrogen native ligand, estradiol, are summarized in Table 1. Also, binding energy, which is another key parameter for assessing ligand–receptor interactions, is also reported. The predicted binding energy values estimated for estradiol were higher than those of BPF. In addition, the amino acid residues involved in the interactions of the ERα –estradiol and ERα–BPF complexes were not identical. The total number of amino acid residues interacting with estradiol was 20, while only 17 amino acid residues exhibited interaction with BPF. Furthermore, only 13 interacting ERα amino acid residues were common between estradiol and BPF. In addition, the hydrogen-bonding interaction observed between the Hie-524 and BPF was not observed. Instead, BPF formed a hydrogen-bonding interaction with Thr-347.

#### 3.1.2. IFD of BPS with ERα

The docking pose of BPS showed that 17 amino acid residues of the ERα were involved in various molecular interactions (Figure 2b). The interacting amino acid residues are Met-343, Leu-346, Thr-347, leu-349, Trp-383, Leu-384, Leu-387, Met-388, Leu-391, Arg-394, Phe-404, Met-421, Ile-424, Phe-425, Leu-428, Leu-525 and Leu-540. Two hydrogen-bonding interactions with Thr-347 and Leu-387 were observed. In addition, one pi–pi interaction with Phe-404 was also observed (Figure 2b).

A comparison of the binding poses of the ERα native ligand in ERα-estradiol complex (Figure 4a) with that of ERα-BPS complex (Figure 2b) indicated that only 14 interacting amino acid residues of ERα were overlapping between estradiol and BPS. Furthermore, the two hydrogen-bonding interactions observed between estradiol and residues Glu-353 and Hie-524 were not seen for BPS (Figure 4a). Instead, BPS formed two different hydrogen-bonding interactions, involving residues Thr-347 and Leu-387 (Figure 2b).

### 3.2. IFD of Bisphenols with ERβ

#### 3.2.1. IFD of BPF with ERβ

The BPF-ERβ docking complex demonstrated extensive interactions with multiple ERβ amino acid residues. In total, 17 residues participated in molecular interactions with BPF, including hydrogen bonds, van der Waals forces, and hydrophobic interactions. The interacting residues were Met-295, Leu-298, Ala-302, Met-336, Leu-339, Met-340, Leu-343, Phe-356, Ile-373, Ile-376, Phe-377, Leu-380, Gly-472, Met-473, Hie-475, Leu-476 and Met-479. A single hydrogen bond was contributed by Hie-475. In addition, one pi–pi interaction by Phe-356 was also observed (Figure 3a).

Similarly, the molecular interactions between the native ligand estradiol and ERβ are depicted in Figure 4b. A total of 22 ERβ residues were involved in interactions with estradiol, including Met-295, Leu-298, Thr-299, Leu-301, Ala-302, Glu-305, Met-336, Leu-339, Met-340, Leu-343, Arg-346, Phe-356, Ile-373, Ile-376, Phe-377, Leu-380, Gly-472, Met-473, Hie-475, Leu-476, Met-479 and Leu-490. In addition, two hydrogen-bonding interactions with Glu-305 and Hie-475 were observed. In addition, one pi–pi interaction with Phe-356 was also observed (Figure 4b).

Additional docking metrics, including the IFD score, Dock score, and Glide score, which are important for evaluating the structural binding behaviour of both BPA analogue, BPF and the estrogen native ligand, estradiol, are summarized in Table 1. Binding energy, another key parameter for assessing ligand–receptor interactions, is also reported. The predicted binding energy values for estradiol were higher than the values estimated for BPF. In addition, the amino acid residues involved in the interactions of ERβ –BPF and ERβ–native ligand complexes were not identical. The total number of amino acid residues interacting with estradiol was 22, whereas only 17 residues interacted with BPF, and all 17 interacting residues were common to estradiol-interacting amino acid residues. Additionally, the hydrogen-bonding interaction observed between estradiol and residue Glu-305 (Figure 4b) was not detected for BPF (Figure 3a).

#### 3.2.2. IFD of BPS with ERβ

The docking pose of BPS showed that 15 amino acid residues of the ERβ were involved in various molecular interactions (Figure 3b). These residues include Met-295, Leu-298, Leu-301, Ala-302, Glu-305, Met-336, Leu-339, Met-340, Leu-343, Arg-346, Phe-356, Ile-373, Ile-376, Phe-377 and Leu-380. In addition, one hydrogen-bonding and two pi–pi interactions were shown by Phe-356. A comparison of the binding poses of the ERβ-BPS complex (Figure 3b) with the native ligand complex ERβ-Estradiol (Figure 4b) indicated 15 ERβ-interacting residues were identified, all of which are shared with estradiol-interacting residues, while estradiol interacts with 22 residues in total. However, hydrogen-bonding interactions displayed by Glu-305 and Hie-475 in ERβ-estradiol complex (Figure 4b) are not observed in ERβ-BPS complex (Figure 3b).

## 4. Discussion

The purpose of this study was to explore the potential endocrine-disrupting activity and evaluate the safety assessment of two commonly used BPA analogues, BPF and BPS, with primary focus on reproductive hormone signalling disruption, specifically estrogen-mediated pathways. The results of structural binding characterization and molecular interactions of these analogues revealed that both bisphenol ligands, BPF and BPS, were placed within the canonical ligand-binding pockets of ERα and ERβ. In addition, the binding pattern and molecular interactions of these analogues in the ligand-binding sites of ERα and ERβ showed some similarity with their native ligand, estradiol. However, in practical terms, these interactions are less able to stabilize the active receptor conformation that drives classical transcriptional signalling, a profile consistent with partial or weak agonism in cellular systems. Further, their docking complex poses are supported by weaker estimated binding energies, which have substantially lower values than the values estimated for native ligands of ERα and ERβ, estradiol. Additional structural binding metrics, including the Dock score, IFD score, and Glide score, estimated for both the bisphenol analogues, also showed lower values than those calculated for ERα and ERβ native ligands, estradiol. Several other factors deemed to contribute greatly to the stability and higher binding energy values of ligand within the binding environment of protein receptor involve chemical interactions, including salt bridges, hydrophobic interactions, van der Waals forces and hydrogen bonding. Although the indicated BPA analogues, BPF and BPS, showed some similarities in how they fit into the ligand-binding pockets of ERα and ERβ, they did not fully match the amino acid environment of the native ligand, estradiol. Therefore, these results suggest that the potential for these bisphenol analogues to compete with the estradiol binding to ERα and ERβ is minimal. Based on the structural binding characterization results, BPF and BPS exhibit lower predicted binding stability to ERα and ERβ compared with the reference ligand (estradiol), suggesting weaker receptor–ligand interactions under the modelled conditions. However, considering the limitations of this study, these findings are preliminary and should not be interpreted as evidence of reduced or absent endocrine activity. The IFD and MM/GBSA analyses provide computational estimates of binding affinity and do not directly evaluate receptor activation, antagonism, or downstream functional effects. In addition, only ERα and ERβ were examined, without consideration of other nuclear or membrane-associated receptors, alternative signalling pathways, or dose- and tissue-specific factors that may influence biological responses in vivo. Therefore, experimental validation through binding assays, transcriptional activity studies, replicate simulations, and complementary in vitro and in vivo investigations is essential to verify and clarify the biological significance of these observations. Future studies should also evaluate known metabolites of the above-mentioned BPA analogues to provide a more comprehensive assessment of receptor binding and potential endocrine activity.

In a wider endocrine context and based on current evidence, endocrine activity cannot be predicted solely from ER behaviour. Even small chemical or stereochemical changes can alter binding patterns through specific residue interactions, thereby modulating subsequent signalling events [37,38]. A similar structure–activity relationship may apply to BPF and BPS, even when their binding to ER is comparatively weak. In addition, thyroid-related mechanisms are also plausible, as docking studies of non-phthalate plasticizers suggest potential TRα binding with energy profiles consistent with low-dose effects. Supporting this view, multi-omics analyses reveal that environmental hormones act through complex, multi-target networks relevant to hepatocellular carcinoma. Collectively, these observations underscore the need to move beyond ER-focused assays when evaluating endocrine risk [9,39]. Contemporary endocrine science also cautions that low and sometimes nonmonotonic exposures can be consequential in pregnancy and early life, which argues against treating “BPA-free” as a proxy for endocrine safety without targeted testing that reflects real-world mixtures and timing of exposure [5,6,7].

Furthermore, reports on BPF and BPS are mixed, with some studies showing no binding, others low binding, and a few suggesting strong binding to estrogen receptors. Overall, the evidence on their estrogenic activity remains inconclusive; for example, a computational study by Usman and Ahmad (2019) found that BPF and BPS did not exhibit strong binding to estrogen receptors [40]. Recently, another computational study indicated that BPF acted as a partial agonist by binding to the estrogen receptor and inducing receptor activation to a lesser extent than full agonists [41]. Conversely, the reviewed literature indicates that BPF and BPS can elicit estrogenic and, in some contexts, anti-estrogenic effects in both in vitro and in vivo systems, largely via estrogen receptor-mediated mechanisms, with potencies often comparable to BPA. According to this review, these findings raise concerns regarding the suitability of BPF and BPS as safer alternatives to BPA [31]. According to a recent study, BPF and BPS activate Atlantic cod estrogen receptors, with BPF showing potency comparable to or higher than BPA, and BPS also exhibiting notable estrogenic activity. These results indicate that both compounds act as estrogenic endocrine disruptors, similar to BPA [42]. In zebrafish (*Danio rerio*), BPF and BPS activated estrogen receptors, with BPF stronger than BPS, demonstrating their estrogenic activity in vivo [43]. Recently, in zebrafish (*Danio rerio*), BPF showed consistent estrogenic activity, whereas BPS exhibited weaker and less consistent effects, indicating some estrogenic potential but inconclusive evidence for BPS [44]. BPF and BPS exhibit weak agonistic activity on human estrogen receptors, with a preference for ERβ [45].

In sum, our structure-based analyses show that BPF and BPS bind ERα and ERβ but with less favourable interaction networks than estradiol, aligning with generally weaker in vitro estrogenicity, although divergent results have also been reported. However, lower ER potency does not imply negligible risk when exposure is widespread and multifactorial. A mixture-aware programme that evaluates parent compounds and major metabolites across ER, AR, and TR, and that spans mechanism-focused assays, targeted animal studies, and well-designed cohorts, offers the most direct path to decision-useful risk estimates for BPA analogues [5,6,7,21,30,31,39].

## 5. Conclusions

The present docking and MM/GBSA analyses indicate that BPF and BPS exhibit lower predicted binding affinities toward ERα and ERβ compared with estradiol, suggesting comparatively weaker receptor–ligand interactions under the computational conditions applied. However, it is important to note that these in silico metrics primarily reflect potential binding tendencies and thermodynamic stability; they do not provide direct evidence of receptor activation, antagonism, or downstream functional signalling. Consequently, caution is warranted when interpreting these results, and no functional conclusions regarding ER signalling or endocrine effects should be inferred solely from docking and MM/GBSA data. To more accurately assess the biological relevance of these findings, experimental validation such as receptor binding assays, transcriptional activity studies, and replicate simulations with clear reporting of computational parameters will be necessary. Moreover, estrogen receptors represent only a subset of potential endocrine-related targets. BPF and BPS may interact with other nuclear or membrane-associated receptors, as well as non-receptor signalling pathways involved in reproductive or developmental processes. Future studies combining in silico, in vitro, and in vivo approaches will be important to evaluate dose-, tissue-, and context-dependent effects and to provide a more comprehensive understanding of the potential biological impact of these bisphenols.

## Figures and Tables

**Table 1:** Structural binding descriptors of BPA analogues (BPF, BPS), and estrogen receptors (ERα, ERβ), native ligand, estradiol.

Receptor	Ligand	Number of Interacting Residues	IFD Score	Docking Score (Kcal/mol)	Glide Score (Kcal/mol)	MMGB-SA (Kcal/mol)
ERα	BPF	17	−535.13	−7.50	−8.55	−46.46
ERα	BPS	17	−534.96	−7.60	−8.11	−48.94
ERα	Estradiol	20	−540.48	−10.82	−10.82	−116.2
ERβ	BPF	17	−516.33	−7.05	−8.10	−48.48
ERβ	BPS	15	−516.11	−8.31	−8.83	−51.23
ERβ	Estradiol	22	−520.69	−11.20	−11.20	−124.00

## Data Availability

The original contributions presented in this study are included in the article. Further inquiries can be directed to the corresponding author.
